# A Retrospective Study on the Impact of COVID-19 on Emergency General Surgery

**DOI:** 10.7759/cureus.29281

**Published:** 2022-09-18

**Authors:** Leo Kretzmer, Ahmed Elmaradny, Murad A Jabir, Rahim Hussain, Maninder Bhambra, Moustafa Mourad, Steven J Robinson, Martin Wadley, Anthony Perry, Mohamed Saad

**Affiliations:** 1 Department of General Surgery, Worcestershire Acute Hospitals NHS Trust, Worcester, GBR; 2 Department of General and UGI (Upper Gastrointestinal) Surgery, Worcestershire Acute Hospitals NHS Trust, Worcester, GBR; 3 Department of Surgical Oncology, South Egypt Cancer Institute, Assiut, EGY; 4 Department of General Surgery, Tanta University Hospital, Tanta, EGY

**Keywords:** conservative management, laparotomies, cholecystectomies, appendicitis, covid 19

## Abstract

Background

The coronavirus pandemic has caused global disruption to all aspects of life. This disturbance has been most notable in the medical world. Political, societal, medical, and behavioral alterations have forced emergency surgical practices to adapt. This study investigated the impact of coronavirus 2019 (COVID-19) at a busy surgical center.

Methodology

This is a retrospective observational study. Three study periods were analyzed: pre-COVID, first wave, and second wave. Data were collected on referrals, diagnoses, investigations, management pathways, outcomes, patient behavior, and consultant practice. A one-way analysis of variance (ANOVA test) was used for the analysis of parametric data and the Mann-Whitney U test for non-parametric data.

Results

Declining numbers of patients presented across the three periods. There was a severe disruption in performing emergency general surgeries during the first wave, propagated by alterations in clinical decision-making, as well as fluctuations in societal and patient behavior. Despite the effects of the second wave being significantly more profound in terms of hospitalization and COVID-related mortality, a paradoxical, gradual return to the norm was noted, which was seen in referral pathways, imaging decisions, and management strategies.

Conclusion

Our data is suggestive of society, both within and outside the medical sphere, adjusting to life with COVID-19.

## Introduction

Coronavirus disease 2019 (COVID-19) has posed a substantial challenge to the global community since it was declared a pandemic by the World Health Organisation (WHO) in March 2020 [1]. Over the past two years, its effects have disrupted almost every walk of life; at an individual level with national lockdowns compromising people’s mental health and livelihoods with 9 million jobs furloughed [2-4] and at a societal level with an economic recession and a gross domestic product decline of 9.7%, the sharpest fall witnessed in recorded history [5,6]. Needless to say, these ramifications have perhaps been most significant in healthcare.

All specialties have faced difficulties during the pandemic either directly or indirectly. Surgical specialties, though not actively involved in managing COVID patients, were nonetheless affected. Reduced elective operations and redeployment of doctors across the National Health Service (NHS) have considerably impacted waiting times, affected training, and career progression [7,8]. Other consequences of the pandemic are only just beginning to be felt, including delayed cancer diagnoses and prolonged waiting lists for elective procedures [9]. This paper follows an initial review published in December 2020 [10] and will further evaluate the effects of COVID-19 in a busy general surgical department into the second wave.

There have been changes in both the presentation and management of emergency surgical cases. Due to concerns about nosocomial COVID-19 infection, bed pressure, lack of access to clean intensive care units (ITU), and guidance from governing bodies, there was a predilection to conservatively manage patients [11]. There was a decline in the frequency of emergency surgical admissions, with a reduction in the length of hospital stay. Patients presenting to the hospital had presented later during the COVID period than before the pandemic [10-13].

At the time of writing, the United Kingdom is documenting record-level new infections due to the omicron variant of coronavirus, which is thought to be three times more infectious than its predecessor: the delta variant [14]. Currently, 82.4% of people over the age of 12 years are double-vaccinated and 59% have had booster vaccinations [15].

The original paper aimed to compare surgical practice during the pre-COVID period and in the first wave. The aim of this study is to primarily review emergency surgical practice during the second wave, to determine if there are differences in the management of surgical emergencies between these three periods and to identify the causative factors that may introduce them.

## Materials and methods

This is a retrospective cohort study. The data were collected from a single consultant surgeon’s on-call take during the second wave of COVID-19 in the United Kingdom, a period between 8-10th and 18-21st of January 2021. This data was then compared to data collected pre-COVID (7-9th and 17-20th of February 2020) and during the first wave (18-21st and 29-31st of May) to give an idea about the difference, if present, in managing various acute surgical conditions during the pre-COVID, first wave, and second wave, respectively.

All patients referred to the general surgical take at Worcester Royal Hospital were considered for inclusion in this study. Patient referrals and sources are recorded on the Surgical Patient Management System (SPMS), a digital database used by the trust to document the referrals, admissions, investigations, and management plan for each patient. SPMS was then used to identify patients from the designated time periods. Exclusion criteria included: no notes available, duplicate entries, patients admitted under other specialties, or re-attendance within one time period for the same diagnosis. A total number of 90 patients were identified for the second wave, 72 of them were eligible for inclusion compared to 112 and 90 in the pre-COVID and first wave, respectively.

Using the virtual clinical databases and workstations, all required data were collected for the above patients. Data were then analyzed in Microsoft Excel (Microsoft Corporation, Redmond, WA). For p-values, one-way analysis of variance (ANOVA) tests were used for parametric data and Mann-Whitney U tests for non-parametric data. We considered p-values <0.05 statistically significant and used a 95% confidence interval as a target.

## Results

Referrals and admissions

The number of admissions declined throughout the pandemic (112 vs. 90 vs. 72; P=0.90533), showing a 20% decrease in hospital admissions in the first wave from pre-COVID as well as between the two waves. Furthermore, there is a noticeable continuous rise in the mean American Society of Anesthesiologists (ASA) score throughout the whole period from 1.89 pre-COVID to 1.98 and 2.11 in both COVID waves, respectively (P=0.326647). Interestingly, the admission/operation ratio between pre-COVID and the second wave was almost the same, 29.5% pre and 28% in the second wave while it was found to be reduced to 23.3% during the first wave (P=0.728211).

**Figure 1:**
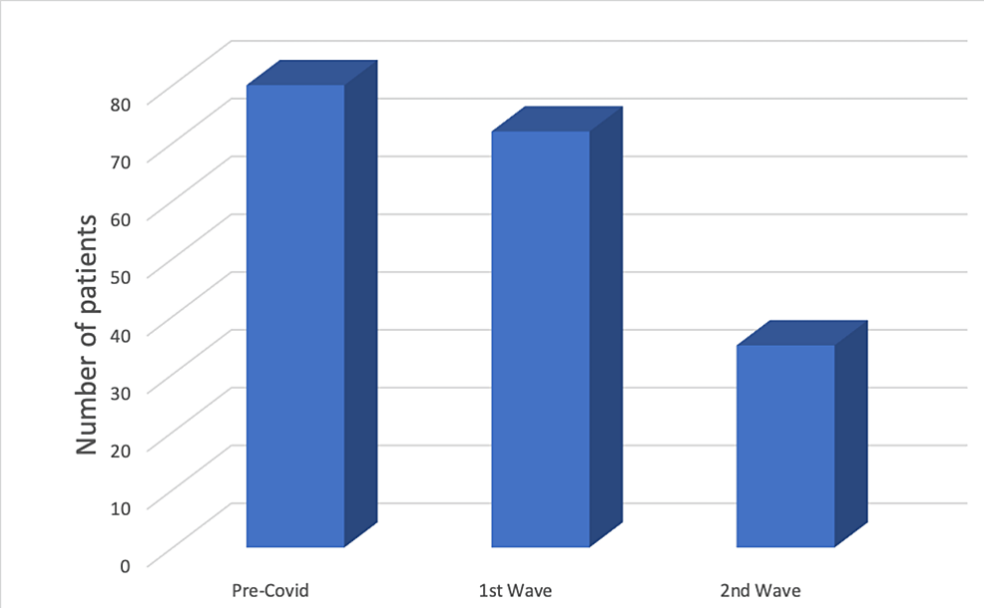
Graph showing the frequency of admissions from Accident and Emergency (A&E)

**Figure 2:**
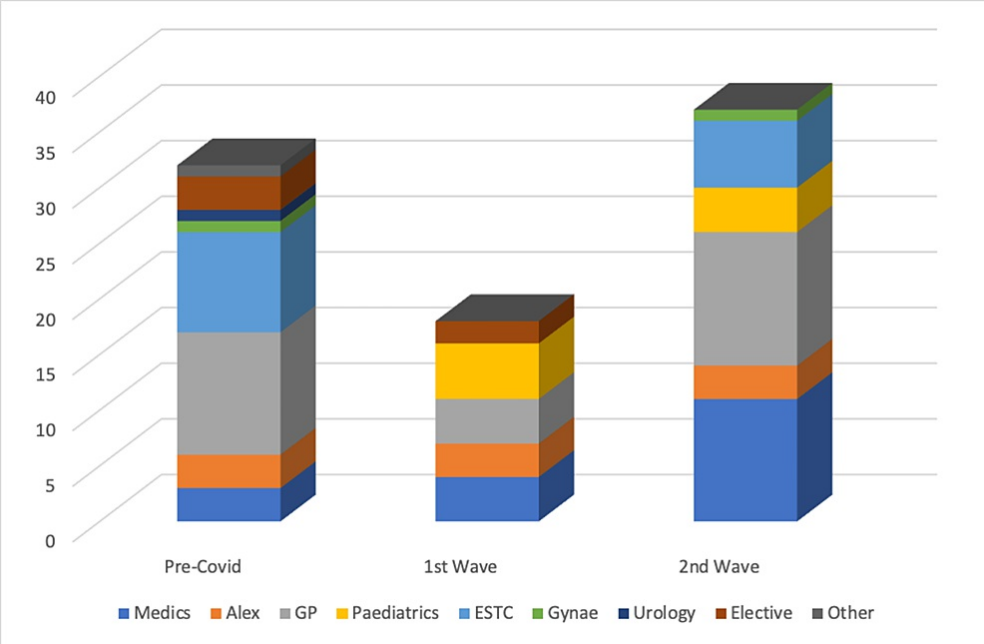
Graph showing routes of admissions excluding Accident and Emergency (A&E)

Accident and Emergency (A&E) was the most frequent route of admission for patients during the pre-COVID period and during the first wave, with 71.43% and 80.00% of patients being referred from there, respectively (Figures 1, 2). However, during the second wave, only 48.61% (n = 35; P=0.000053) of patients were referred via A&E.

The emergency surgical treatment clinic (ESTC), which acts as a semi-emergency ambulatory assessment clinic accounted for 8.03% (n = 9) of admissions prior to COVID, however, during the first wave, there were no admissions via ESTC at all. This trend appears to have reversed, as, during the second wave, 8.33% (n = 6; P=0.020289) of referrals were from there.

Likewise, referrals from general practitioners (GP) also reduced from pre-COVID to the first wave (n = 4 vs. 11). As with ESTC, this trend also reversed, showing a threefold rise in GP referrals from the first to second waves (n = 4 vs. 12, respectively; P=0.020289).

Diagnoses

**Figure 3:**
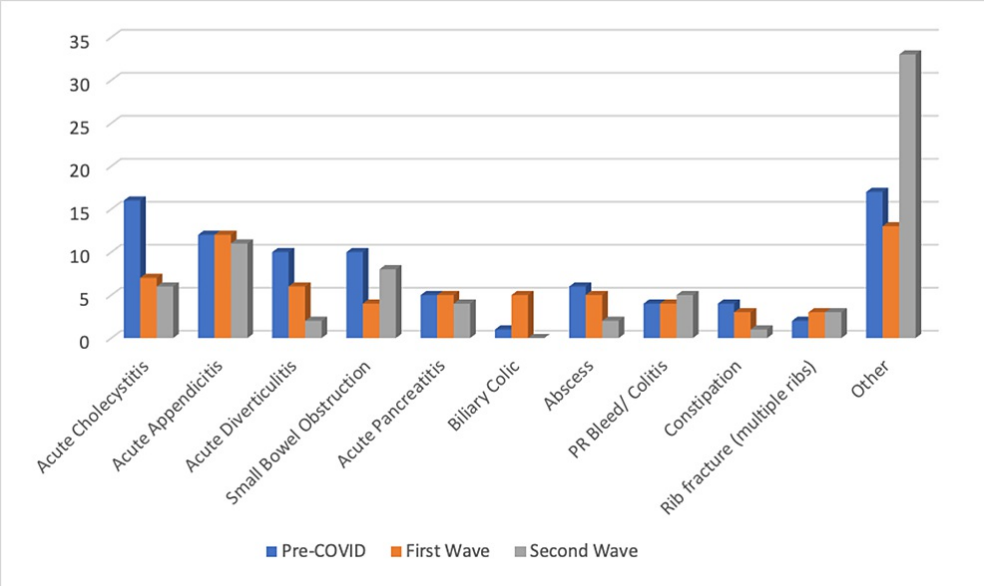
Graph showing frequency of diagnoses encountered over the three periods

Interestingly, the incidence of some conditions (Figure 3) remained constant, namely, acute appendicitis (n = 12, 12, 11; P=0.196894) and pancreatitis (n = 5, 5, 4; P=0.549304), whereas some other conditions appeared to decline. Acute cholecystitis was the most common reason for admission before COVID. The frequency of patients presenting with this dropped by 56% in the first wave, and this decline continued into the second wave (n = 16, 7, 6; P=0.340195).

**Figure 4:**
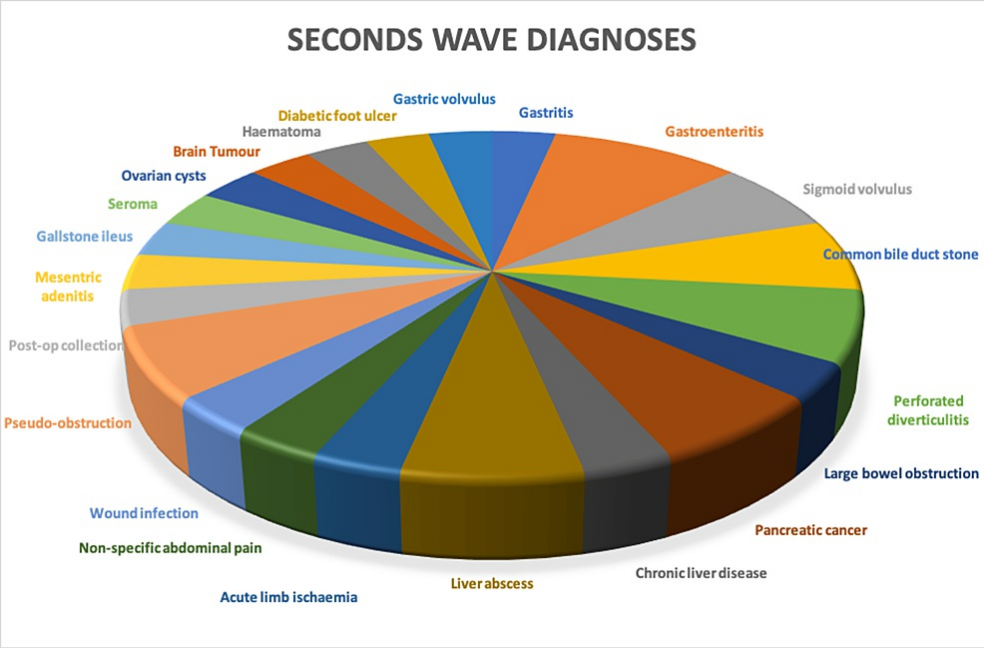
Pie chart demonstrating 'other' second wave diagnoses

Patients in the second wave demonstrated a more disparate range of conditions. In the pre-COVID and first wave, patients presenting with less common diagnoses accounted for 15.18% and 14.44%, respectively. In the second wave, this increased to 45.83% (n = 33; P=0.001247) (Figure 4).

Investigations

**Figure 5:**
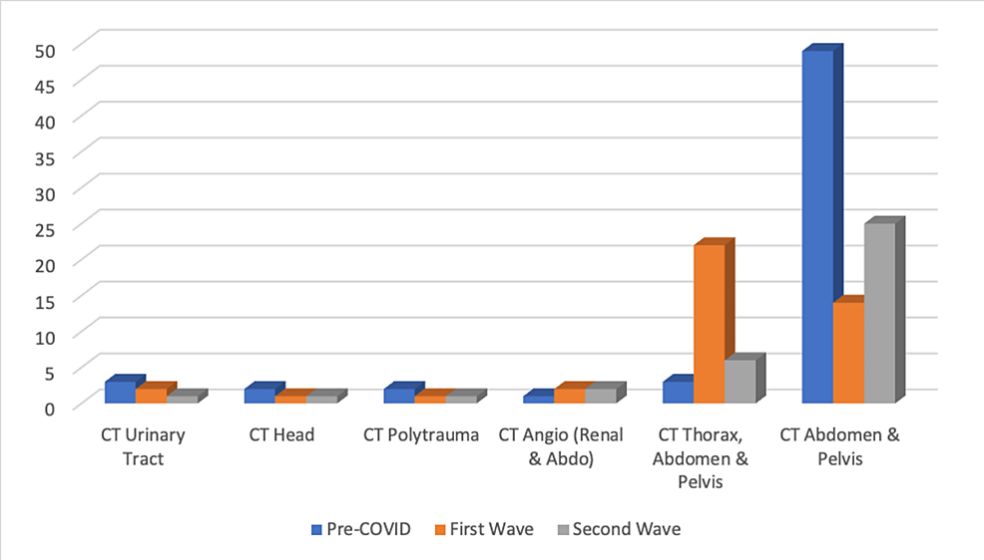
Graph showing the frequency of CT protocols

The number of computed tomography (CT) scans (Figure 5) decreased throughout the three periods (n = 60, 42, 36); however, the proportion of patients who underwent a CT declined in the first wave, and then partially recovered in the second (53.6, 46.6, 50.0%; P=0.730176). A similar fraction of patients in all three waves had CT imaging protocols of the abdomen and pelvis (AP) or the thorax, abdomen, and pelvis (TAP). In the pre-COVID period, there was a clear predilection for CT AP (43.45%; n = 49), whereas, in the first wave, there was a shift toward including the thorax (CT TAP: 52.38%; n = 22; P<0.00001). The second wave showed a limited return to pre-COVID practice. Other CT protocols remained relatively constant and at a low frequency.

**Figure 6:**
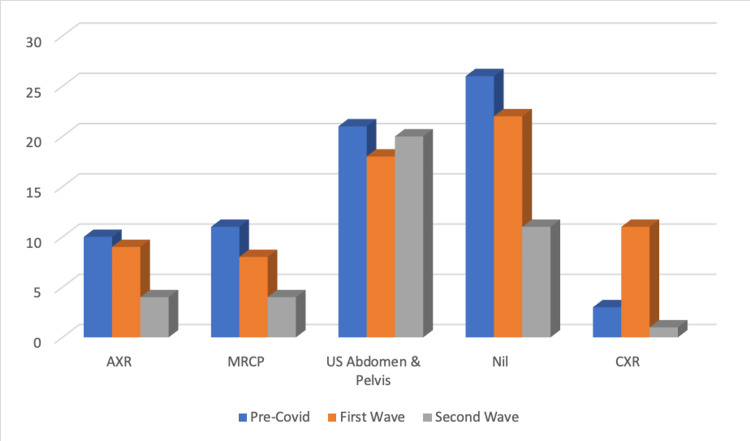
Graph demonstrating the frequency of other imaging modalities

The frequency of other modalities is demonstrated in Figure 6. Abdominal X-rays (AXR) and magnetic resonance cholangiopancreatography (MRCP) scans both declined in frequency throughout the period, (AXR: n = 10, 9, 4; P=0.430187; MRCP: n = 11, 8, 4; P=0.367797). Understandably, the number of chest X-rays (CXR) increased in the first wave, this, however, declined in the second to below pre-COVID levels (n = 3, 11, 1; P=0 .003239). In the first two periods, a similar number of patients did not warrant imaging, 23.21% and 24.44%, respectively. In the second wave, however, only 15.28% (n = 11) were not imaged (P=0.12171).

Management

**Figure 7:**
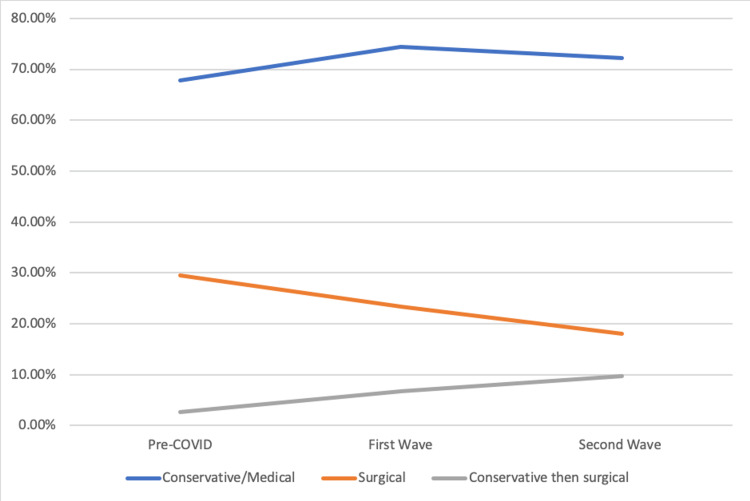
Graph showing the type of management

The initial management of patients is demonstrated in Figure 7. As would be expected, the proportion of patients being managed nonoperatively increased from pre-COVID to the first wave, which then declined slightly into the second wave (67.86% vs. 74.44% vs. 72.22%, respectively; P=0.004197). Throughout the three periods, there was a decline in initially managing patients surgically (n=33 vs. n=21 vs. n=13; P=0.200397). From the pre-COVID period to the second wave, there was a threefold rise in patients initially having medical or conservative management going on to require surgery (2.68 to 9.72%; P=0.035548).

**Figure 8:**
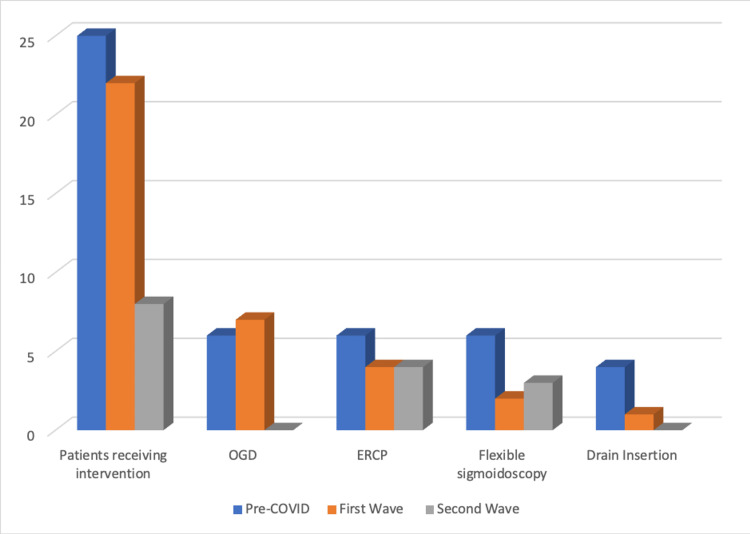
Graph showing the types and frequency of nonoperative interventions throughout the study periods

The proportion of patients who underwent nonoperative interventions was alike in the pre-COVID period and the first wave (n = 25 vs. 22; 22% vs. 24%; P=0.105179). The second wave, however, showed a significant decrease with only 5.56% (n = 8; P=0.105179) undergoing nonoperative intervention (Figure 8). No patients were offered oesophagogastroduodenoscopy (OGD) or drain insertion during the second wave (P=0.08633). Endoscopic retrograde cholangiopancreatography (ERCP) and flexible optical sigmoidoscopy (FOS) remained relatively constant (ERCP: n = 6, 4, 4, P=0.855093; FOS: n = 6, 2, 3, P=0.432619).

Operations

**Figure 9:**
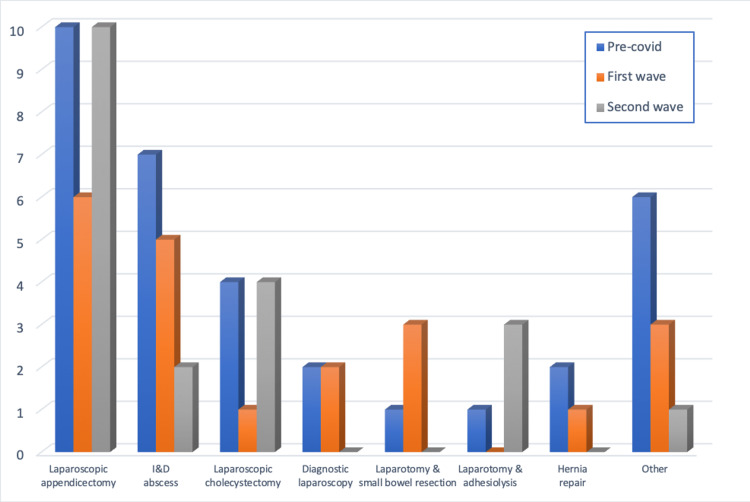
Frequency of the types of operations throughout the investigation period

Appendicectomy was the most frequent operation, and its frequency remained relatively constant throughout the three observation periods (n = 10, 7, 10; P=0.248357) (Figure 9). Laparoscopic cholecystectomy was offered on emergency operating lists in the pre-COVID period while this practice was markedly reduced in the first wave, numbers appear to have reverted by the second wave (n = 4, 1, 4; P=0.339565). The most notable decrease was seen in incision and drainage (I&D) of abscesses (n = 7, 5, 2; P=0.428405).

In the pre-COVID period, where both laparoscopic and open approaches could have been considered, 69.23% (n = 18/25) of cases were managed laparoscopically. In the first wave, this decreased slightly to 62.50% (n = 10/16). However, in the third wave, despite an equivocal number of operations being performed, 82.35% of operations were laparoscopic (n = 14/17). Overall, this trend was not statistically significant (P=0.360315). Only one appendicectomy was performed open during the first wave.

Surgical outcomes

As demonstrated in Table 1, the length of stay (LoS) of medically or conservatively managed patients, which had decreased in the first wave, markedly increased in the second wave (6.37 vs. 2.40 days). Patients managed surgically demonstrated the converse of this from pre-COVID into the first and second waves (5.61, 8.29, 4.77 days).

**Table 1:** Demonstrating LoS and complications for patients admitted in the observed period LoS: length of stay; ITU: intensive care unit

Initial Management		Pre-COVID				First wave				Second wave			
		n	Mean LoS (d)	Mean level of complication (Clavian-Dindo)	Mean ITU stay (d)	n	Mean LoS (d)	Mean level of complication (Clavian-Dindo)	Mean ITU stay (d)	n	Mean LoS (d)	Mean level of complication (Clavian-Dindo)	Mean ITU stay (d)
Conservative		76	4.95 (SD=9.55)			67	2.40 (SD=3.74)			52	6.37 (SD=7.17)		
Surgical	All surgical	33	5.61 (SD=8.22)	2	3	21	8.29 (SD=9.67)	1.5		13	4.77 (SD=4.50)	1.5	
	Without complications	27	4.30 (SD=6.84)			15	4.93 (SD=6.34)			18	5.67 (SD=6.64)		
	With complications	8	11.50 (SD=11.78)	2	3	6	16.67 (SD=11.98)	1.5		2	8.00 (SD=8.49)	1.5	
Conservative then surgical		3	19.33 (SD=15.18)	2	3	4	6.25 (SD=4.19)			7	8.00 (SD=4.16)	2	

## Discussion

The COVID-19 pandemic has caused profound disruption to the NHS and specifically to emergency surgical care. While multiple factors contributed to this disturbance, it can be due to changes in decision-making, societal and patient behavior.

The average numbers of new cases, hospitalizations, and COVID-related deaths in the data collection periods are shown in Table 2. In terms of impact, the second wave of COVID-19 was significantly worse than the first. It would, therefore, be sensible to predict that behaviors and practices picked up in the first wave would be continued or even amplified into the second wave. Our data, however, has instead demonstrated a clear trend of disruption into the first wave and then a partial recovery in the second. This is seen in the number of patients referred to the acute take from both GPs and ESTC clinics, the frequency of common procedures performed, and the amount of chest imaging.

**Table 2:** COVID-19 statistics pertaining to data collection periods Data from the UK Government

	Average Daily New Cases	Average New Daily Hospitalizations	Average Daily Deaths
First wave (18 – 21/05/20) (29 – 31/05/20)	2089	733	221
Second wave (8 – 10/01/21) (18 – 21/01/21)	39044	3996	1185

We saw a decrease in admissions from GPs or semi-urgent ambulatory care in the first wave, but this recovered in the second. This may be due to GP closures, public lockdowns, and changes in patient behavior contributing to patients' delayed presentation or seeking medical attention via alternate routes. In the second wave, it appears there was a shift back to normality.

Guidance was released suggesting that acute cholecystitis should be managed non-operatively if possible [11]. This may have contributed to the decline in the frequency of laparoscopic cholecystectomies performed. Our center normally tries to offer cholecystectomies for patients with acute cholecystitis within seven days of diagnosis when possible, a practice that was avoided during the first wave but seemingly restored in the second.

In the first wave, there was a tendency toward including the chest in patients undergoing abdominal CT imaging. In patients not requiring a CT scan, a much higher proportion had CXRs performed. However, in the second wave, it appears these practices had faded. This seems to fall in line with guidance: The Royal College of Radiologists (RCR) released guidance on April 30, 2020, recommending the inclusion of CT chest in patients already undergoing abdominal imaging [16]. In June, due to the practice having a low diagnostic yield, this was rescinded [17].

Whilst decisions pertaining to CT imaging may vary in line with institutional judgments, the frequency of CXRs may be viewed as a litmus test of the individual clinicians’ concern, as CXRs are easy to arrange and pose relatively little risk of radiation exposure to the patient. We saw a significant increase of CXRs performed in the first wave, an increase that then declined into the second wave (n = 3, 11, 1). The uptake in the first wave presumably reflects the constant thought that was given to COVID-19 due to the national lockdown, perpetual media attention, and a sense of apprehension and fear associated with it. Interestingly, in the second wave, only one CXR was performed despite being at the zenith of the pandemic’s severity.

Similarly, the first wave saw an increase in the proportion of patients initially managed conservatively, possibly reflecting a hesitation of surgeons to primarily operate due to apparent risks. These may have included perceived risks of COVID, scarcity of staff due to re-deployment, or a lack of access to operating theaters or intensive care beds postoperatively to name a few. Regardless of what these factors were, they were evidently alleviated somewhat by the second wave, as there was a statistically significant reduction in patients being initially conservatively managed in this period. Interestingly, throughout the three periods, there was a steady increase in the number of patients who failed non-operative management and then required surgical intervention.

The frequency of patients presenting with diagnoses of acute appendicitis and pancreatitis remained constant throughout the three observation periods. Whilst the pandemic’s effects on society were widespread, it appears it did not significantly alter any causative or preventative factors for these conditions. In Western countries, acute appendicitis has an incidence of 5.7 - 50 patients per 100,000 inhabitants per year, which was maintained in our patient population throughout the pandemic [18]. Conversely, we saw a decline in patients presenting with acute cholecystitis, non-perforated diverticulitis, abscesses, and constipation in both the first and second waves despite the incidence of these conditions presumably remaining constant. While these numbers are low and not statistically significant, it does suggest that there may have been an element of patient and clinician behavior influencing this. Patients with these conditions may have elected to delay presentation due to apprehension about being in a hospital environment. Similarly, following assessment in A&E, surgeons may have opted to discharge patients with oral antibiotics to avoid admission and potential nosocomial COVID-19 infection. This change is also reflected in reduced numbers of patients being admitted to hospitals throughout the pandemic.

The patients that were admitted became more unwell further into the pandemic with the mean ASA of patients presenting gradually increasing. This may be a result of avoiding or delaying presentation to the hospital.

This study is a small-scale, single-center, retrospective study, and statistical significance on several variables was difficult due to low numbers.

## Conclusions

This was a single-center, retrospective observational study looking at how COVID-19 has impacted emergency surgical care. We have demonstrated a trend of severe disruption in the first wave, propagated by alterations in clinical decision-making, as well as fluctuation in societal and patient behavior. Despite the effects of the second wave being significantly more profound, we have observed a gradual return to the norm. This data is likely evidence of society, both inside and outside the medical sphere, adjusting to life with COVID-19.
